# Epoxy-Based Interlocking Membranes for All Solid-State Lithium Ion Batteries: The Effects of Amine Curing Agents on Electrochemical Properties

**DOI:** 10.3390/polym13193244

**Published:** 2021-09-24

**Authors:** Tsung-Yu Yu, Shih-Chieh Yeh, Jen-Yu Lee, Nae-Lih Wu, Ru-Jong Jeng

**Affiliations:** 1 Institute of Polymer Science and Engineering, National Taiwan University, Taipei 106, Taiwan; d07549003@ntu.edu.tw (T.-Y.Y.); so850306w@gmail.com (J.-Y.L.); 2 Advanced Research Center for Green Materials Science and Technology, National Taiwan University, Taipei 106, Taiwan; 3 Department of Chemical Engineering, National Taiwan University, Taipei 106, Taiwan

**Keywords:** amino/epoxy, solid state electrolyte (SPE), all-solid-state lithium ion battery (ASSLIBs), interlocking bilayer

## Abstract

In this study, a series of crosslinked membranes were prepared as solid polymer electrolytes (SPEs) for all-solid-state lithium ion batteries (ASSLIBs). An epoxy-containing copolymer (glycidyl methacrylate-co-poly(ethylene glycol) methyl ether methacrylate, PGA) and two amine curing agents, linear Jeffamine ED2003 and hyperbranched polyethyleneimine (PEI), were utilized to prepare SPEs with various crosslinking degrees. The PGA/polyethylene oxide (PEO) blends were cured by ED2003 and PEI to obtain slightly and heavily crosslinked structures, respectively. For further optimizing the interfacial and the electrochemical properties, an interlocking bilayer membrane based on overlapping and subsequent curing of PGA/PEO/ED2003 and PEO/PEI layers was developed. The presence of this amino/epoxy network can inhibit PEO crystallinity and maintain the dimensional stability of membranes. For the slightly crosslinked PGA/PEO/ED2003 membrane, an ionic conductivity of 5.61 × 10^−4^ S cm^−1^ and a lithium ion transference number (tLi^+^) of 0.43 were obtained, along with a specific capacity of 156 mAh g^−1^ (0.05 C) acquired from an assembled half-cell battery. However, the capacity retention retained only 54% after 100 cycles (0.2 C, 80 °C), possibly because the PEO-based electrolyte was inclined to recrystallize after long term thermal treatment. On the other hand, the highly crosslinked PGA/PEO/PEI membrane exhibited a similar ionic conductivity of 3.44 × 10^−4^ S cm^−1^ and a tLi^+^ of 0.52. Yet, poor interfacial adhesion between the membrane and the cathode brought about a low specific capacity of 48 mAh g^−1^. For the reinforced interlocking bilayer membrane, an ionic conductivity of 3.24 × 10^−4^ S cm^−1^ and a tLi^+^ of 0.42 could be achieved. Moreover, the capacity retention reached as high as 80% after 100 cycles (0.2 C, 80 °C). This is because the presence of the epoxy-based interlocking bilayer structure can block the pathway of lithium dendrite puncture effectively. We demonstrate that the unique interlocking bilayer structure is capable of offering a new approach to fabricate a robust SPE for ASSLIBs.

## 1. Introduction

Lithium ion batteries (LIB) became the mainstream portable energy because of the boost energy requirement for electric vehicles (EV) [1,2,3,4,5]. For safer lithium ion batteries, solid polymer electrolytes (SPEs) have a decisive role owing to their non-flammability and there being no leakage risk in the practical operation [6,7,8,9,10,11]. Polyethylene oxide (PEO) based electrolytes act as the most attractive material in solid polymer electrolytes because the ethylene oxide (EO) units of PEO possess chain flexibility, high dielectric constant, and strong Li^+^-ion solvating ability [12,13]. In fact, the ionic conductivities of typical PEO-based electrolytes are capable of reaching a range of 10^−4^–10^−3^ S cm^−1^ [6]. However, poor mechanical properties and dimensional instability increase the risk of a short circuit phenomenon [14]. Therefore, several versatile SPEs were developed to solve these problems, such as composited polymer electrolytes [15,16], biosource-derived electrolytes [17,18], UV-cured electrolytes [19,20,21], and thermal crosslinked electrolytes [22,23]. Among these SPEs, the incorporation of a crosslinking network in the polymer matrix is one of the useful methods to block lithium dendrite crossover to the opposite electrode [24,25]. Primary amines containing precursors, especially Jeffamine, are of great interest for obtaining crosslinking networks in robust SPE matrices. This is due to the fact that only mild reaction conditions are required for amino groups to respectively react with carboxylic acid, epoxide, anhydride, etc., and the presence of EO units in Jeffamine would certainly enhance the solubility of the Li salt [26].

Indeed, several attempts to utilize ether-containing amine curing agents for developing crosslinked epoxy polymers as SPEs have been reported. In 2018, Lopez et al. developed a dual crosslinked SPEs from adipic acid and Jeffamine, which provides robust mechanical properties while maintaining ionic conductivity of ~10^−5^ S cm^−1^ at 80 °C [27]. In 2020, Li et al. prepared comb-chain crosslinker-based SPEs using poly(glycidyl methacrylate) (PGMA) and diamine poly(ethylene glycol) via amino/epoxy reaction. As a result, a high ionic conductivity (1.31 × 10^−4^ S cm^−1^) at 40 °C was obtained [28]. In addition, Lehmann et al. prepared a polyethyleneimine (PEI)/epoxy network exhibiting an ionic conductivity of 3.9 × 10^–5^ S cm^−1^ at 40 °C [29]. It is important to note that a highly crosslinked network would bring about better bulk properties and reduced crystallinity. Yet, the addition of 20 wt% plasticizer was required for achieving high ionic conductivity. In 2019, Aldalur and coworkers reported a flowable polymer electrolyte based on poly(ethylene-alt-maleic anhydride) and Jeffamine M-2070 as a buffer layer between Li metal electrode to improve the cycling stability of 76% after 70 cycles [30]. In 2020, Fedeli et al. developed so-called nanocomposite solid polymer electrolytes based on octakis(3-glycidyl-oxypropyldimethylsiloxy)octasilsesquioxane and Jeffamine ED-2003, exhibiting an ionic conductivity of ~10^−4^ S cm^−1^ at 80 °C [7]. Based on the above, the formation of slightly crosslinked matrices by utilizing linear amino compounds as curing agents for epoxy resins would provide a high ionic conductivity. To further improve the electrochemical stability and the bulk properties, a hyperbranched amino compound such as PEI was introduced to increase crosslinking density in the membranes [29,31]. However, the trade-off of ionic conductivity and bulk properties has to be taken into account by adding an appropriate amount of plasticizer or liquid electrolyte. Therefore, the preparation of an optimized polymer network in a membrane comprising the advantages derived from the above-mentioned two systems would be a sensible approach.

**Figure 1:**
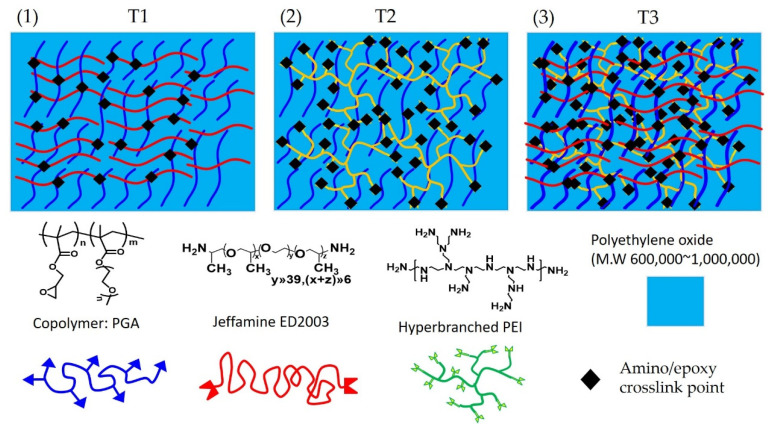
Three types of SPEs: (**1**) slightly crosslinked SPE based on ED2003, (**2**) tightly crosslinked SPE based on hyperbranched PEI, (**3**) an interlocking bilayer structure of SPE.

In this study, an epoxy containing copolymer (PGA) comprising glycidyl methacrylate (GMA) and poly(ethylene glycol) methyl ether methacrylate (PEGMA) along with a certain amount of PEO was cured with a linear diamine ED2003 and a hyperbranched PEI, respectively. As shown in Figure 1, three types of SPEs were investigated. Type 1 SPE is a slightly crosslinked SPE consisting of PGA/PEO cured by the linear ED2003, whereas type 2 SPE is a heavily crosslinked SPE comprising PGA/PEO cured by the hyperbranched PEI. In addition, type 3 SPE is an interlocking bilayer membrane based on overlapping and subsequent curing of PGA/PEO/ED2003 and PEO/PEI layers. This provided an optimized polymer network in a membrane exhibiting desirable bulk properties and cycle stability. The crystallinity and the thermal properties of these membranes were firstly investigated by polarized optical microscopy (POM) and differential scanning calorimeter (DSC). Electrochemical impedance spectroscopy (EIS) was utilized to estimate the ionic conductivity and the transference number (tLi^+^) of membranes. A half-cell structure of Li°|SPE|LiFePO_4_ (LFP) was utilized as a platform to compare the reliability of these SPE membranes.

## 2. Experimental

### 2.1. Materials

Linear curing agent Jeffaimne ED2003 was obtained from Huntsman corporation (Woodlands, TX, USA). Poly(ethylene glycol) methyl ether methacrylate (PEGMA, M_n_ = 500) monomer, glycidyl methacrylate (GMA, 97%) monomer, hyperbranched polyethyleneimine (PEI, Mn = 800), and lithium salt of bis(trifluoromethane)sulfonimide lithium salt (LiTFSI) were purchased from Sigma-Aldrich and used as received. Poly(ethylene oxide) (PEO) (M.W = 600,000~1,000,000) was provided by Acros (Geel, Belgium). Azobisisobutyronitrile (AIBN) initiator was purchased from Aencore (Melbourne, Australia). Anisole and acetonitrile (ACN) were used without further purification. Cathode material, lithium iron phosphate (LiFePO_4_; LFP), and lithium (Li) metal for the anode for lithium ion batteries (LIBs) were obtained from UBIQ Technology Corp. (Taipei, Taiwan), which were stored under vacuum to avoid humidity.

### 2.2. Preparation of Epoxy Containing Copolymer

**Scheme 1:**
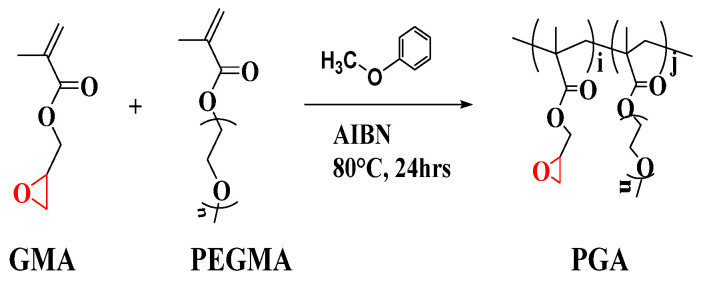
Synthetic route of GMA-co-PEGMA.

The synthetic route for PGA copolymer is shown in Scheme 1. A mixture of GMA (2.843 g, 0.02 mol) and PEGMA (10 g, 0.02 mol) in 30 mL anisole was added into a three-neck bottom flask, followed by adding AIBN (0.433 g, 0.0002 mol) as the initiator. The solution was stirred at 80 °C for 24 h under nitrogen atmosphere. After the reaction, the product was precipitated in n-hexane and then dried under vacuum. The number average molecular weight of PGA was measured to be 46,000 with a polydispersity of 2.4 as investigated by gel permeation chromatography (GPC; Figure S1).

### 2.3. Preparation of SPE Membranes

Three types of membranes consisting of an amino/epoxy crosslinked matrix were prepared as SPEs. Firstly, the PEO was dissolved in acetonitrile to form a 4 wt% solution. For type 1 SPE, PGA and ED2003 with a 1:1 stoichiometric ratio were mixed homogeneously at room temperature to form GAE. Various amounts of the GAE solution were added to the PEO solution with a range of weight ratios (PEO/GAE) including 100:0, 80:20, 70:30, 50:50, and 30:70.

**Scheme 2:**
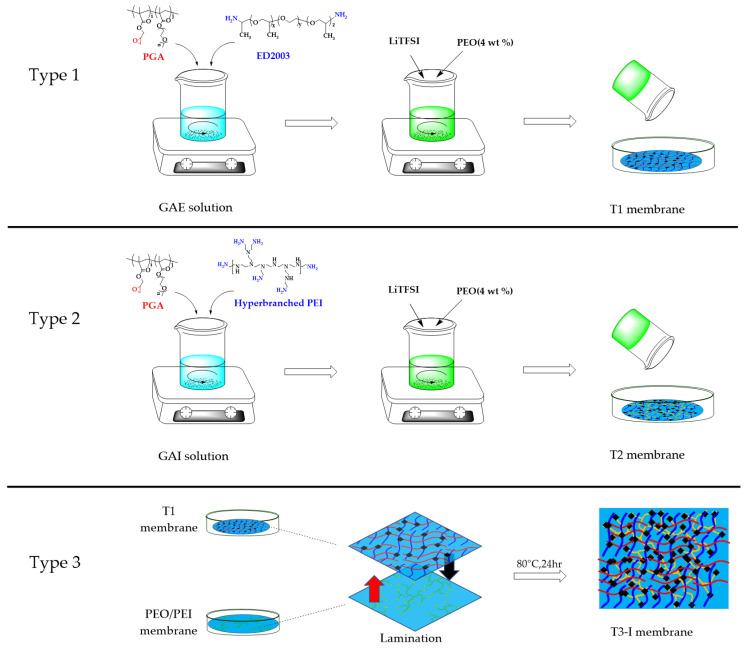
Preparation processes of SPE membranes.

The designed SPEs with PEO and GAE were abbreviated as T1-х (х is a weight ratio of GAE in SPE). Finally, the solutions were cast on a Teflon plate and dried in an oven at 80 °C for 24 h to obtain SPE membranes (Scheme 2). For type 2 SPE, PGA and PEI with a 1:1 stoichiometric ratio were mixed homogeneously at room temperature to form GAI. The GAI solution was mixed with the PEO solution (4 wt%). The mixed solution was subsequently cast on a Teflon plate to form a membrane in the same manner as the T1 series. The type 2 SPE was abbreviated as T2-х (х is a weight ratio of GAI in SPE). Apart from that, the type 3 SPE was an interlocking bilayer structure prepared from overlapping of T1-20 and PEO/PEI membranes. The PEO/PEI membrane was prepared by casting PEI-containing PEO solution on a Teflon plate, followed by thermal treatment at 60 °C for 24 h. These two membranes were laminated and then thermally treated at 80 °C for 24 h. As a result, the interlocking SPE membrane, T3-I, was obtained. The thicknesses of all membranes were controlled at 200 ± 10 μm with the same solid content per unit area. It is important to note that a certain amount of LiTFSI with a ratio of EO/Li (15:1) was added to each SPE membrane.

### 2.4. Material Characterization

The copolymer (PGA) was dissolved in deuterated chloroform for ^1^H NMR analyses on AVIII HD 400 MHz (Bruker, MA, USA), and the molecular weight was measured by gel permeation chromatography (GPC, model: S2020, KM3 Scientific, Taipei, Taiwan) using DMF as the mobile phase with a flow rate of 0.8 mL min^−1^ and polystyrene (PS) as the standard for calibration. The glass transition temperature (T_g_) of the SPE was measured by the differential scanning calorimeter (DSC, Model: Q20, TA Instruments, New Castle, DE, USA) with a scan rate of 10 °C min^−1^. Surface morphology images of these SPE samples was obtained on a polarized optical microscope (POM, Model: BX53, Olympus, Tokyo, Japan).

### 2.5. Electrochemical Characterization

Ionic conductivity (*σ*) of the SPEs in this work was evaluated by electrochemical impedance spectroscopy (EIS) at temperatures from 20 °C to 80 °C (with an interval of 20 °C). The SPE membrane for testing was sandwiched between two stainless steel (SS) electrodes in the CR2032 coin cell with CH Instruments (Model: CH-614D). The cell gap between two SS electrodes was fixed by a 200 μm polypropylene (PP) spacer. The bulk impedance of the SPE was measured by the AC impedance method at the amplitude of 10 mV, and the frequency window was from 1 MHz to 1 Hz. The ionic conductivity was determined by Equation (1):

(1) $$\sigma =\frac{d}{R_{b}\cdot A}$$

where *σ* is lithium ionic conductivity (S cm^−1^), *d* is the thickness of SPE membranes, *A* is the area of SPE membranes (cm^2^), and *R_b_* is the bulk impedance of SPE (Ω) [32,33,34].

The electrochemical stability window of SPEs was investigated by the linear sweep voltammetry (LSV) method. The SPE membrane was sandwiched between Li metal and SS and placed in CR2032 (Li°|SPE|SS). The scanning potential voltage was from open circuit potential (OCP) to 6 V (vs. Li^+^/Li) at a rate of 0.2 mV/s at 80 °C [35]. The electrochemical compatibility of SPEs was investigated for testing the long-term lithium plating/striping cycles by a Neware electrochemical workstation (BTS-4000). Li symmetric cell (Li°|SPE|Li°) was polarized at a current density of 0.1 mA cm^−2^. The duration of each cycle was 1 h, and polarization voltage was recorded (a point per minute) [30].

The lithium ion transference number (tLi^+^) was defined from Bruce–Vincent–Evans’ Equation (2):

(2) $${\mathrm{tLi}}^{+}=\frac{I_{s}\left(\Delta V-I_{0}R_{0}\right)}{I_{0}\left(\Delta V-I_{s}R_{S}\right)}$$

where *I_0_, I_s_, R_0_*_,_ and *R_s_* are initial DC current, steady-state DC current, initial interfacial resistances, and steady state resistances, respectively [36]. At first, the SPE sample was sandwiched in the CR2032 (Li°|SPE|Li°) and kept at 80 °C for 24 h to equilibrate before measurement. The measurement was set to a DC polarization voltage at ∆*V* equal to 10 mV. The *R_0_* was measured with EIS in advance at the amplitude of 10 mV over a frequency from 1 MHz to 1 Hz. Subsequently, the *I_s_* was measured after polarization for 6000 s. Finally, the *R_s_* was again determined on the same condition of *R_0_*. Furthermore, the thickness dependence of tLi^+^ was also investigated in the range of 100 μm to 600 μm, measured by Mitutoyo thickness gage. Three samples of each membrane were measured for the average normalized tLi^+^.

All-solid-state batteries consisted of LFP as the cathode, lithium metal as the anode, and the SPE membrane sandwiched between two electrodes. All the cells were assembled in an argon-filled atmosphere. Charge and discharge tests of the batteries were performed using the CT3001 workstation (LANHE). The active material mass loading of LFP was 11.4 mg cm^−2^ (in diameter of 13 mm), and the lithium metal was cut in a diameter of 15 mm. The SPE membrane with the thickness of 200 µm was cut into a disk having a diameter of 18 mm. The charge–discharge cycling was carried out at current rates from 0.05 C to 1.0 C over a voltage range from 2.5 V to 4.2 V at 80 °C.

## 3. Results and Discussion

### 3.1. Synthesis and Characterization

PGA copolymer was synthesized via free radical polymerization according to the literature [37]. The chemical structure of this copolymer was investigated by ^1^H NMR (Figure S2). The ratio of PEGMA/GMA in PGA copolymer was 3:1, as calculated by integration of the protons from the methyl group at 3.38 ppm and the glycidyl group at 3.22 ppm, which is similar to the feeding ratio.

**Figure 2:**
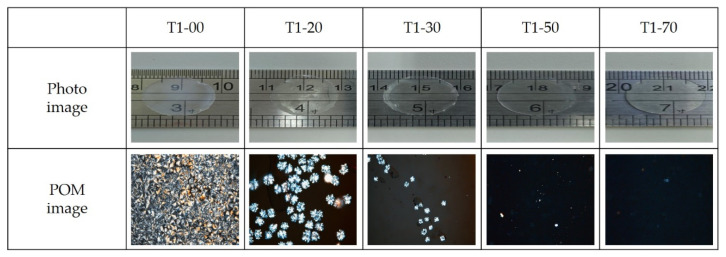
Photo and POM images of T1 series SPE membranes.

**Figure 3:**
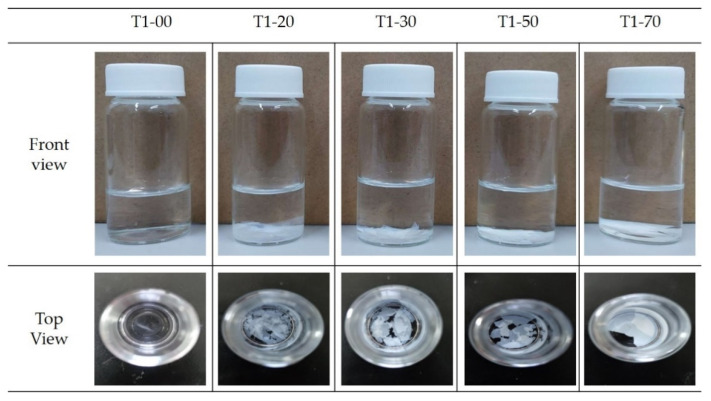
Solvent resistance of SPE membranes.

Photo and POM images of SPE membranes are shown in Figure 2. The membrane without GAE network, i.e., cured PGA/ED2003 in the matrix (T1-00), was opaque due to the presence of PEO crystallinity. As illustrated in the POM image, the microcrystals were well dispersed in the matrix. The membrane became transparent when the GAE network was incorporated, indicating the crystallization of EO segment was hindered in the crosslinked matrix, i.e., the presence of the GAE network. As depicted in POM images, the crystallinity decreased with increasing content of the GAE network. Especially, the crystallinity disappeared for the T1-50 and the T1-70 membranes. To investigate the effects of crosslinking on the SPE membranes, solubility tests in acetonitrile were conducted at room temperature for 48 h. In Figure 3, only the T1-00 sample was completely soluble in acetonitrile due to the absence of crosslinked structure. On the other hand, T1-20, T1-30, T1-50, and T1-70 samples exhibited solvent resistance properties due to the presence of the GAE network in the SPE membranes.

### 3.2. Thermal Properties of SPE Membranes

**Figure 4:**
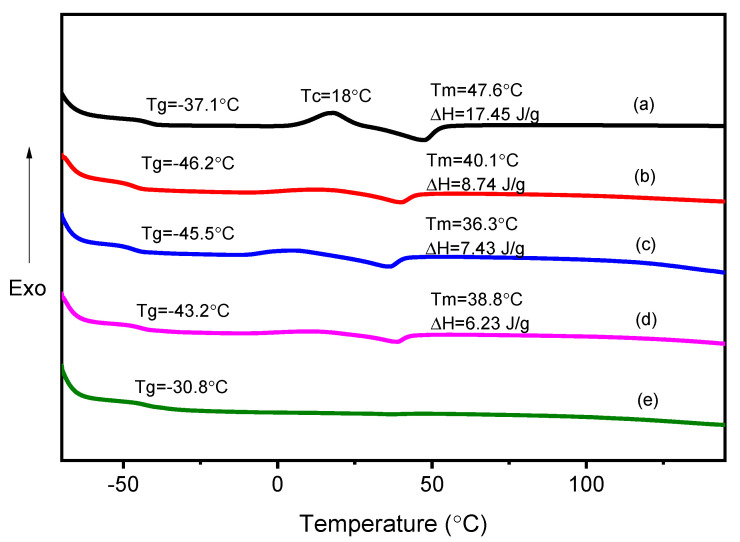
DSC thermograms: (**a**) T1-00, (**b**) T1-20, (**c**) T1-30, (**d**) T1-50, and (**e**) T1-70.

Thermal transitions of the T1-x samples were investigated by DSC in the range of −70 to 150 °C at a scanning rate of 10 °C min^−1^. In Figure 4, distinct T_g_ (−37.1 °C), recrystallization temperature (T_c_; 18 °C), and melting temperature (T_m_; 47.6 °C) were observed for the T1-00 sample. However, no distinct T_c_ was observed for the crosslinked T1-20, T1-30, T1-50, or T1-70 samples. Moreover, the ∆H (T_m_) value decreased with an increasing degree of crosslinking for these samples. It is important to note that no T_m_ peak was obverted for the T1-70 sample. This indicated that the extensive amino/epoxy crosslinked networks could effectively suppress the re-arrangement of EO segments. This is consistent with the results in the POM study.

### 3.3. Lithium Ion Conductivity (σ) and Lithium Ion Transference Number (tLi^+^)

**Figure 5:**
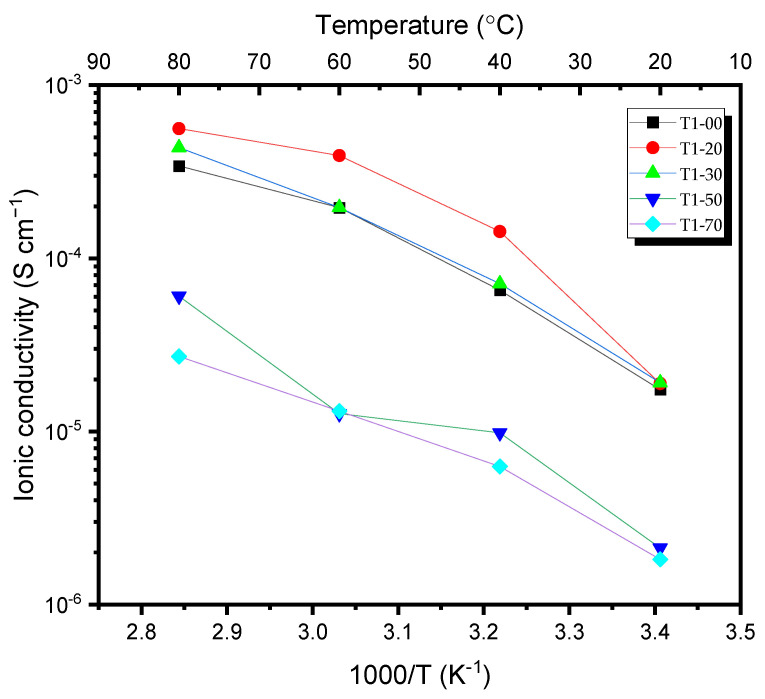
Temperature-dependent ionic conductivities of T1 series SPE membranes.

Ionic conductivity plays a critical role in the realization of the membrane for SPEs in LIBs, which was investigated by EIS. Ionic conductivities of the T1 series membranes at different temperatures are shown in Figure 5. T1-00, T1-20, and T1-30 exhibited similar ionic conductivities at room temperature. Moreover, a much better ionic conductivity of 5.61 × 10^−4^ S cm^−1^ was obtained for T1-20 when the temperature was raised to 80 °C. This also indicates that the abundant EO soft segments of ED2003 and PGA allowed the smooth migration of lithium ion (Li^+^) in the membrane matrix [26]. However, the ionic conductivities of T1-50 and T1-70 were significantly declined to 6.05 × 10^−5^ and 2.71 × 10^−5^ S cm^−1^ (80 °C), respectively. The results indicate that an optimized content of crosslinked amino/epoxy network is required for the balance of bulk and electrical properties.

Lithium ion transference number (tLi^+^) is defined as the ratio of the electric current derived from the cation to the total electric current [36,38]. Generally, a higher tLi^+^ indicates that more Li^+^ cations are dominant in the ion conducting performance. Moreover, a large tLi^+^ can reduce concentration polarization of electrolytes during charge–discharge steps and realize a higher power density [39]. The tLi^+^ values of SPEs are often calculated by Bruce–Vincent–Evans’ equation. The transference number of PEO electrolyte (T1-00) is reported in literature [40], which is significantly influenced by the lithium salt concentration and the PEO molecular weight [41]. In fact, poor mechanical properties of the PEO electrolyte often cause dimensional instability at high temperatures. In this study, thickness dependence of tLi^+^ for T1-00 was firstly investigated (Table 1). The highest tLi^+^ of 0.72 was achieved when the thickness of the thermally treated T1-00 membrane was 50 μm. In addition, the tLi^+^ values were 0.551 and 0.322 for the treated samples with thicknesses of 200 μm and 350 μm, respectively. It is important to note that the thicknesses of T1-00 membranes decreased drastically when the membranes were assembled and stored at 80 °C for hours. On the contrary, the crosslinked T1-20 exhibited no dimensional change after the thermal treatment. The tLi^+^ of T1-20 with a thickness of 225 μm was estimated to be 0.43 (Figure S3). Based on the above, the tLi^+^ would increase with decreasing thickness of the SPE membrane, which is consistent with that reported in literature [42].

### 3.4. Electrochemical Stability and Compatibility of SPEs

**Figure 6:**
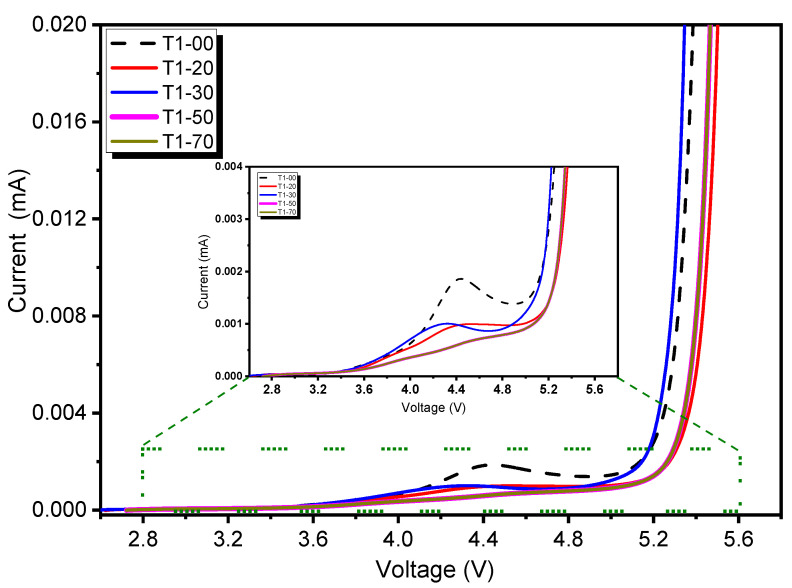
Linear sweep voltammetry (LSV) measurements of T1 series SPE membranes.

The linear sweep voltammetry (LSV) method was utilized for investigating the oxidation potential of the SPE membrane. As seen in Figure 6, the T1-00 exhibited an obvious oxidation peak at 4.4 V with a leakage current of 0.002 mA. This was caused by the oxidation of EO segments in the SPE membrane [30]. Moreover, the leakage currents of T1-20 and T1-30 were decreased to 0.001 mA at 4.4 V. For T1-50 and T1-70 samples, no obvious oxidation peaks were observed with degradation onsets shifting to 5.0 V. This indicated that the incorporation of the crosslinked network in the membrane was effective to enhance the electrochemical stability of the membranes.

**Figure 7:**
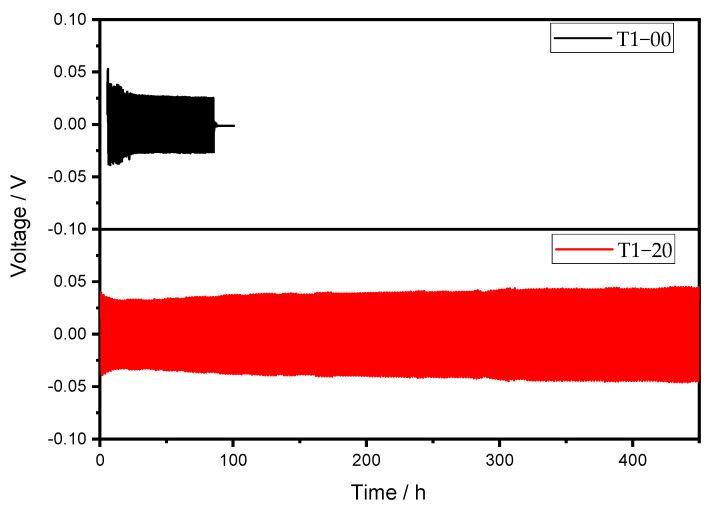
Strip-plate tests on Li°|SPE|Li° symmetric cells employing T1-00 and T1-20 at 80 °C.

To study the interface stability between the SPE and the Li metal, strip-plate tests on Li°|SPE|Li° symmetric cells were carried out on the SPEs at 80 °C and a current density of 0.1 mA cm^−2^ (Figure 7). For the T1-00 test, a short circuit was observed after 130 h. This implied the presence of the dendrite crossover during the lithium plating/stripping process. On the other hand, no short circuit was observed for the T1-20 sample during cycling over 450 h. This may be attributed to the formation of a favorable solid electrolyte interface on the lithium metal [27]. The amino/epoxy networks would suppress dendrite crossover during long-term cycling and prevent short circuit occurrence.

### 3.5. Characterization of Li°|SPE|LFP Half-Cells

**Figure 8:**
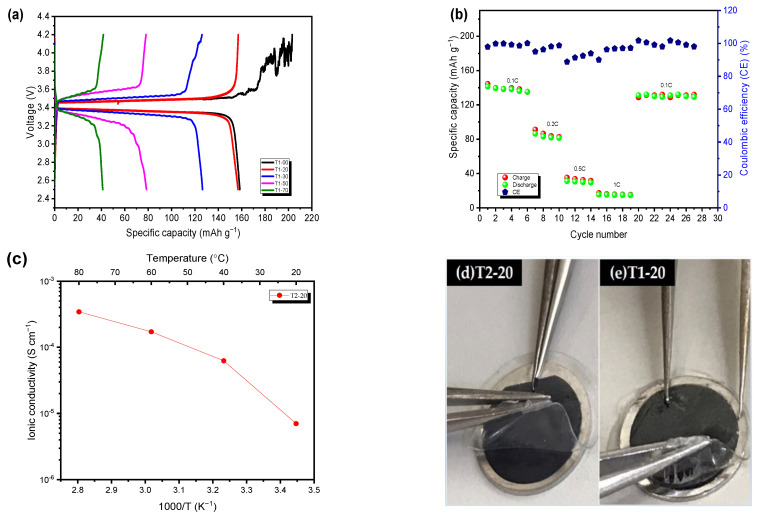
(**a**) The charge−discharge profiles using T1-00, T1-20, T1-30, T1-50, and T1-70 as SPE at 80 °C (0.05 C). (**b**) The specific capacity and the Coulombic efficiency at different C rates in cycle tests for Li°|T1-20|LFP cells at 80 °C. (**c**) Temperature-dependent ionic conductivities of T2-20 and the photos of (**d**) adhesion between T2-20 and cathode, (**e**) adhesion between T1-20 and cathode.

To evaluate the feasibility of the SPE membranes in LIBs, a half cell was assembled using LFP as a cathode and Li metal as an anode. The charge–discharge curves of Li°|T1|LFP at 0.05 C (80 °C) for T1-00, T1-20, T1-30, T1-50, and T1-70 are shown in Figure 8a. The cell with T1-00 exhibited its highest discharging specific capacity of 158 mAh g^−1^. However, a short circuit caused by the formation of lithium dendrites occurred at high voltage. For the cell with T1-20, a specific capacity of 156 mAh g^−1^ was achieved, as illustrated in the charge–discharge profile. This was due to the presence of the advantageous electrochemical properties such as high ionic conductivity, fast migration of Li^+^, and good electrochemical interfacial stability, as mentioned in previous sections. In addition, the rate capability of the cells was recorded at various C rates (Figure 8b). The cell with T1-20 exhibited reversible specific capacities of 140, 86, 31, and 15 mAh g^−1^ at 0.1 C, 0.2 C, 0.5 C, and 1 C, respectively. A high efficiency of capacity recovery was observed when the current density was returned to 0.1 C. Apart from that, the rate capability of the cells with T1-30, T1-50, and T1-70 were also recorded at various C rates, as shown in Figures S4–S6. The feasibility of implementing SPE for rechargeable ASSLIBs was further evaluated by the cycling performance of the Li°|SPE|LFP cell at 80 °C. For the tested cell based on T1-20, the specific capacity retained only 54% of its initial capacity after 100 cycles. Despite that, the sample exhibited reasonably good electrochemical properties in the aspects of ionic conductivity, tLi^+^, and specific capacity. To further improve the cycling stability, a hyperbranched amino compound, PEI, was introduced to replace ED2003 for the amine/epoxy network in the membrane [29,31]. The amine/epoxy network comprised PGA and PEI with a 1:1 stoichiometric ratio. Therefore, a T2-20 SPE based on 20 wt% of PGA/PEI and 80 wt% of PEO was prepared in the same manner as T1-20. In Figure 8c, the T2-20 exhibited the ionic conductivity of 3.44 × 10^−4^ S cm^−1^ at 80 °C, which was close to that of T1-20 (5.61 × 10^−4^ S cm^−1^). Apart from that, tLi^+^ of the T2-20 SPE membrane was measured to be about 0.52 (Figure S7). However, the tested cell based on T2-20 exhibited a specific capacity of 48 mAh g^−1^ at 0.1 C (Figure S8), which was much lower than that of the T1-20 cell. This was due to the poor adhesion between T2-20 and the cathode [44]. As shown in Figure 8d, the T2-20 membrane could be easily peeled off from the cathode without residue material after a charge–discharge test, whereas good interfacial adhesion was maintained between T1-20 and the cathode (Figure 8e). This result manifests that the specific capacity depends not only on ionic conductivity and lithium ion transference number but on the interfacial compatibility as well.

**Figure 9:**
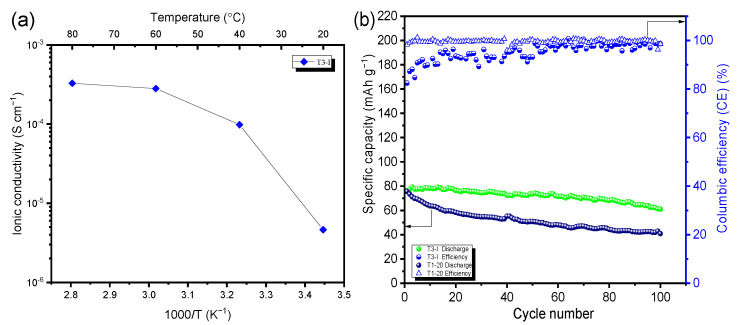
(**a**) Ionic conductivities for T3-I, (**b**) the discharge capacity and the Coulombic efficiency for the Li°|T3-I|LFP and Li°|T1-20|LFP at 80 °C (0.2 C).

For providing an optimized polymer network in a membrane with desirable bulk properties and cycle stability, an interlocking bilayer was developed, namely the T3-I SPE membrane. The T3-I membrane was prepared by overlapping and subsequent curing of the PGA/PEO/ED2003 SPE membrane and the PEO/PEI SPE membrane. T3-I possessed a similar ionic conductivity of 3.24 × 10^−4^ S cm^−1^ to those of T1-20 and T1-00 at 80 °C (Figure 9a) along with a tLi^+^ of 0.42 (Figure S9). Furthermore, the initial capacity of the tested cell based on T3-I was 80 mAh g^−1^ at 0.2C, which was exactly the same as that of the T1-20 cell (Figure 9b). After 50 cycles, the capacity retention was 95% with a Coulombic efficiency of 100%. In addition, the cycle retention was kept at 80% after 100 cycles. It is important to note that the capacity retention of LFP|T1-20|Li was 80% after 50 cycles and further dropped to 54% after 100 cycles. The cell based on the interlocking bilayer membrane indeed exhibited enhanced cycle stability while maintaining comparable electrochemical properties.

## 4. Conclusions

A series of crosslinked SPE membranes were successfully prepared. The membranes comprising the amino/epoxy networks were made of the epoxide-containing PGA cured with a hyperbranched amine (PEI) and a linear amine (ED2003), respectively, in a PEO matrix. The T1-20 membrane consisting of linear ED2003 exhibited an ionic conductivity of 5.61 × 10^−4^ S cm^−1^ and a tLi^+^ of 0.43. Moreover, a high specific capacity of 145 mAh g^−1^ at 0.1 C (80 °C) was achieved for the T1-20 cell. However, that cycle retention was only 54% after 100 cycles. To further improve the cycling stability, a hyperbranched amino compound, PEI, was intended to replace ED2003 for forming a rather tightly crosslinked network in the membrane. However, poor interfacial adhesion was present between SPE and cathode, resulting in a low capacity. Therefore, an interlocking bilayer was developed to obtain a membrane with desirable bulk properties and cycle stability. The reinforced interlocking bilayer membrane, T3-I, presented an ionic conductivity of 3.27 × 10^−4^ S cm^−1^ and a tLi^+^ of 0.42. In addition, the specific capacity retained 80% of its original value (80 mAh g^−1^) after 100 cycles at 0.2 C for the T3-I cell. It is concluded that the design of the interlocking bilayer membrane is effective to achieve a robust SPE for ASSLIBs.

## Figures and Tables

**Table 1:** Thickness-dependent lithium ion transference numbers (tLi^+^) of SPEs.

	Original Thickness *^a^ (*μm)	Thickness *^a,b^* μm	tLi^+^ (80 °C) *^a^*	SPE Type	Ref.
T1-00	100 ± 15	50 ± 15	0.73 ± 0.06	pristine PEO	this work
	200 ± 40	124 ± 40	0.62 ± 0.03	pristine PEO	this work
	300 ± 15	158 ± 15	0.54 ± 0.05	pristine PEO	this work
	400 ± 40	220 ± 40	0.55 ± 0.02	pristine PEO	this work
	500 ± 25	350 ± 25	0.32 ± 0.04	pristine PEO	this work
T1-20	225 ± 30	225 ± 30	0.43 ± 0.03	crosslinked SPE	this work
SPE-PEO	80	-	0.21	pristine PEO	[15]
PEO	200	-	0.36	pristine PEO	[14]
PEO@HPU	150	-	0.46	crosslinked SPE	[43]
PGMA−PEG	100	-	0.234	crosslinked SPE	[28]

*^a^* Deviation was calculated from three samples. *^b^* Thickness after thermal treatment at 80 °C.

## Data Availability

Not applicable.

## Supplementary Materials

The following are available online at https://www.mdpi.com/article/10.3390/polym13193244/s1, Figure S1: Gel permeation chromatography (GPC) analysis of PGA (DMF as solvent) (blue line is a calibration curve, red line is a sample curve and green line is baseline), Figure S2: ^1^H-NMR spectrum of PGA, Figure S3: Chronoamperometry profile of the symmetric Li°|T1-20|Li° (inset shows the EIS curves before and after polarization), Figure S4: Specific capacity and Coulombic efficiency vs. cycle number for the Li°|T1-30|LFP cells at 80 °C, Figure S5: Specific capacity and Coulombic efficiency vs. cycle number for the Li°|T1-50|LFP cells at 80 °C, Figure S6: Specific capacity and Coulombic efficiency vs. cycle number for the Li°|T1-70|LFP cells at 80 °C, Figure S7: Chronoamperometry profile of the symmetric Li°|T2-20|Li° (inset shows the EIS curves before and after polarization), Figure S8: Specific capacity and Coulombic efficiency vs. cycle number for the Li°|T2-20|LiFePO4 cells at 80 °C, Figure S9: Chronoamperometry profile of the symmetric Li|T3-I|Li (inset shows the EIS curves before and after polarization).
