# Supplementary material for: Epoxy-Based Interlocking Membranes for All Solid-State Lithium Ion Batteries: The Effects of Amine Curing Agents on Electrochemical Properties
Source: Polymers (Basel). 2021 Sep 24;13(19):3244. doi: 10.3390/polym13193244 (PMC8513100; doi:10.3390/polym13193244)
Supplement: Supplementary file 1 [file polymers-13-03244-s001.zip › polymers-1380499-supplementary.pdf]

Supporting Information

Epoxy-based Interlocking Membranes for All Solid-State Lithium Ion Batteries: The Effects of Amine Curing Agents on Electrochemical Properties

Tsung-Yu Yu,[1] Shih-Chieh Yeh,[1,2] Jen-Yu Lee,[1] Nae-Lih Wu,[2,3]* Ru-Jong Jeng[1,2]**

1. Institute of Polymer Science and Engineering, National Taiwan University, Taipei 106, Taiwan
2. Advanced Research Center for Green Materials Science and Technology, National Taiwan University, Taipei 106, Taiwan
3. Department of Chemical Engineering, National Taiwan University, Taipei 106, Taiwan

Figure S1. Gel permeation chromatography (GPC) analysis PGA.
Figure S2. ^{1}H-NMR spectrum of PGA
Figure S3. Chronoamperometry profile of the symmetric Li°|T1-20|Li° (inset shows the EIS curves before and after polarization).
Figure S4. Specific capacity and Coulombic efficiency vs. cycle number for the Li°|T1-30|LFP cells at 80 ^{o}C.
Figure S5. Specific capacity and Coulombic efficiency vs. cycle number for the Li°|T1-50|LFP cells at 80 ^{o}C.
Figure S6. Specific capacity and Coulombic efficiency vs. cycle number for the Li°|T1-70|LFP cells at 80 ^{o}C.
Figure S7. Chronoamperometry profile of the symmetric Li°|T2-20|Li° (inset shows the EIS curves before and after polarization).
Figure S8. Specific capacity and Coulombic efficiency vs. cycle number for the Li°|T2-20|LFP cells at 80 ^{o}C.
Figure S9. Chronoamperometry profile of the symmetric Li|T3-I|Li (inset shows the EIS curves before and after polarization).

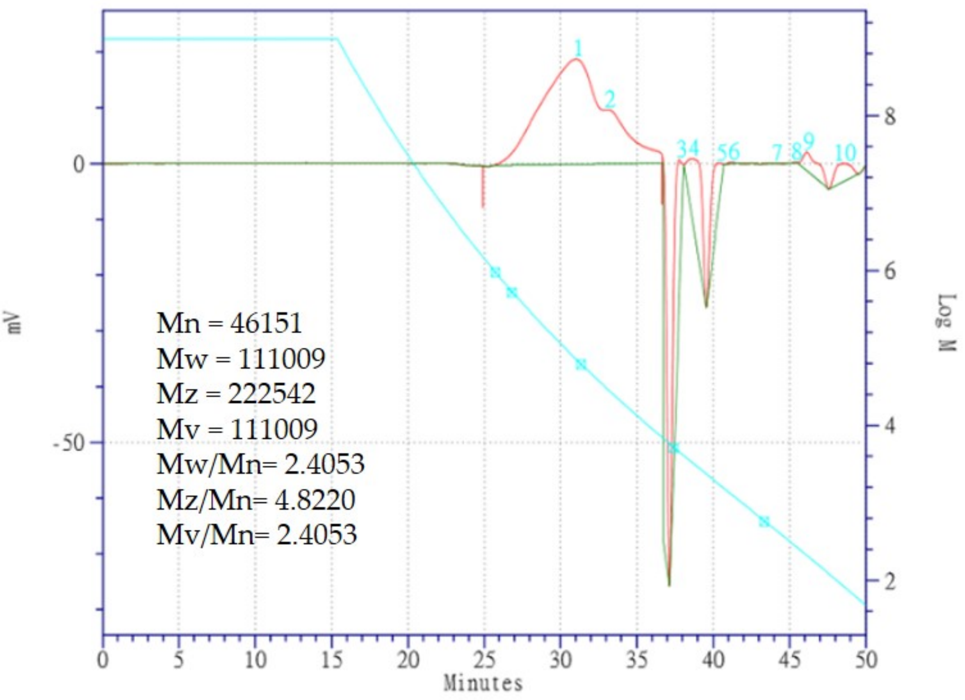

Figure S1. Gel permeation chromatography (GPC) analysis of PGA (DMF as solvent) (blue line is a calibration curve, red line is a sample curve and green line is baseline).

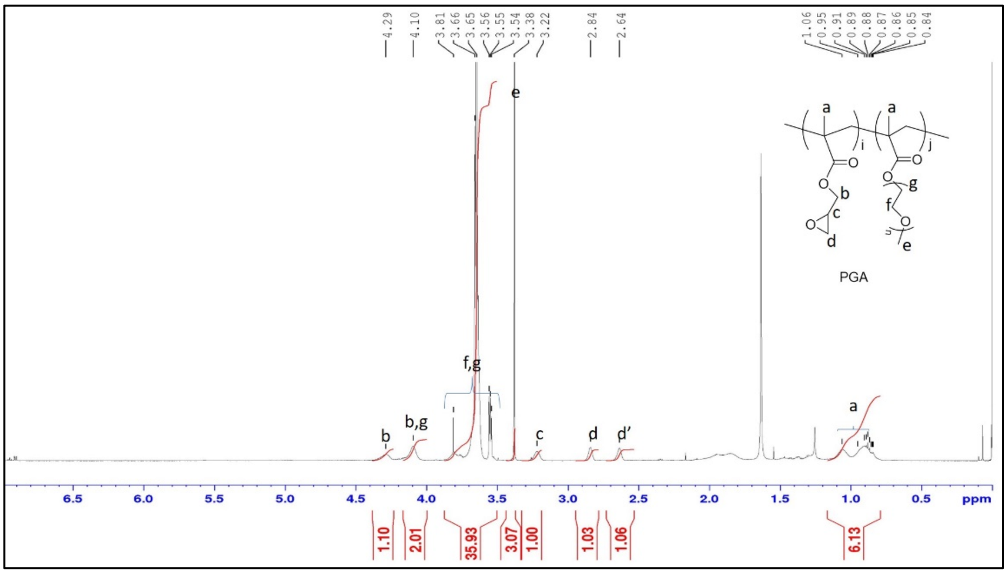

Figure S2 ^{1}H-NMR spectrum of PGA

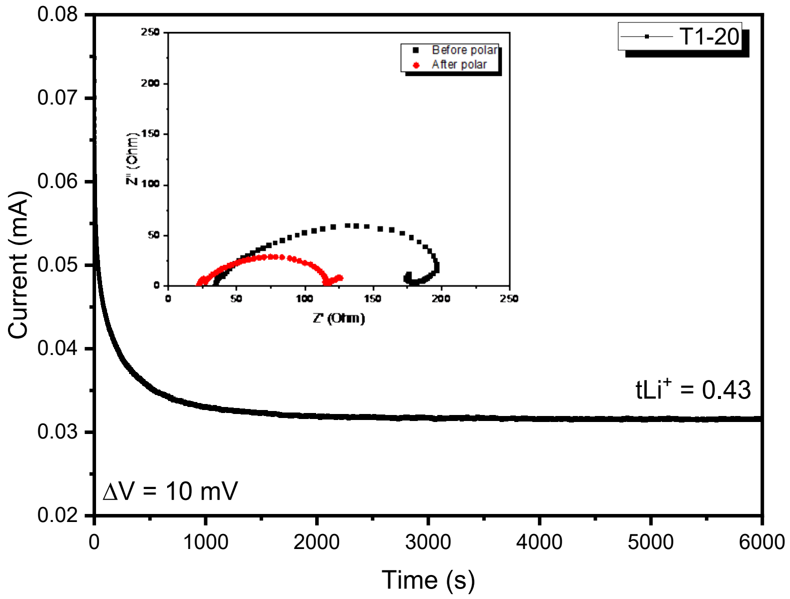

Figure S3. Chronoamperometry profile of the symmetric Li°|T1-20|Li° (inset shows the EIS curves before and after polarization).

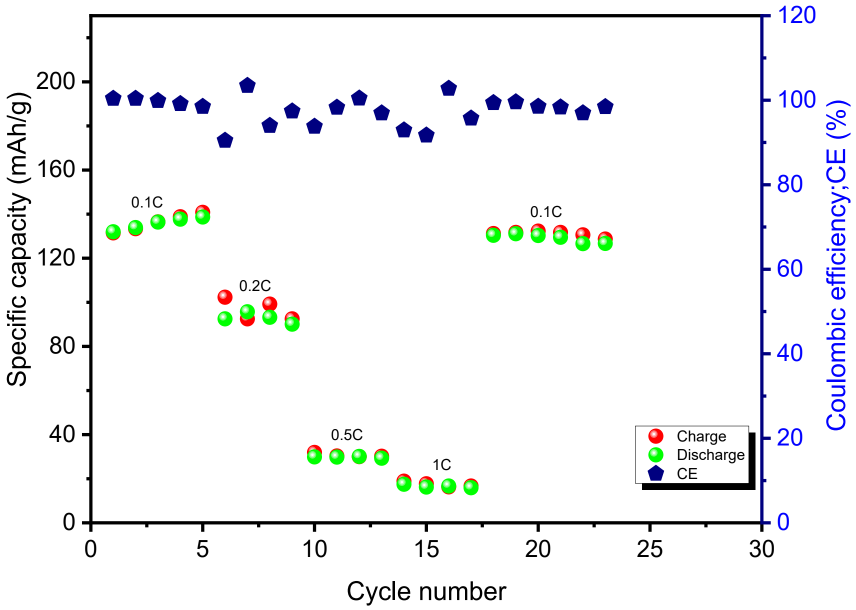

Figure S4. Specific capacity and Coulombic efficiency vs. cycle number for the Li°|T1-30|LFP cells at 80 °C.

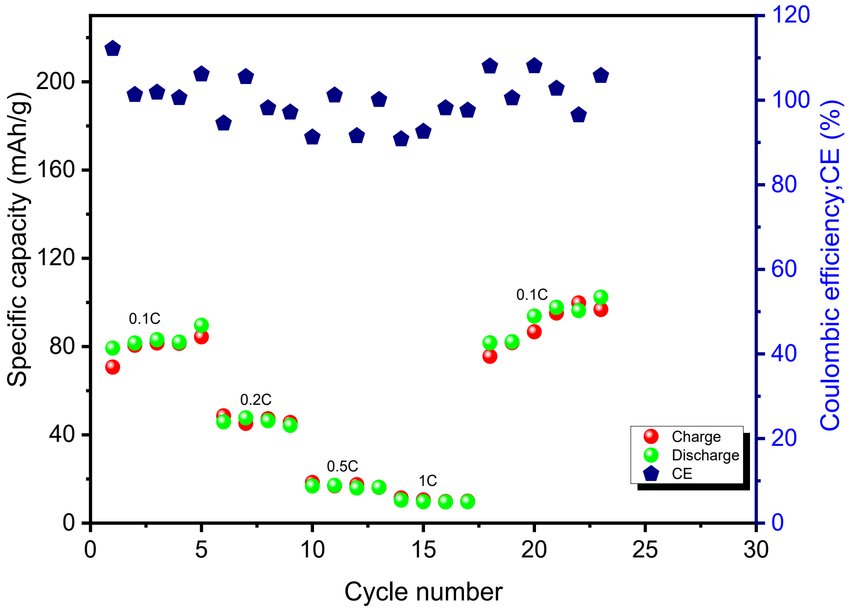

Figure S5. Specific capacity and Coulombic efficiency vs. cycle number for the Li°|T1-50|LFP cells at 80 °C.

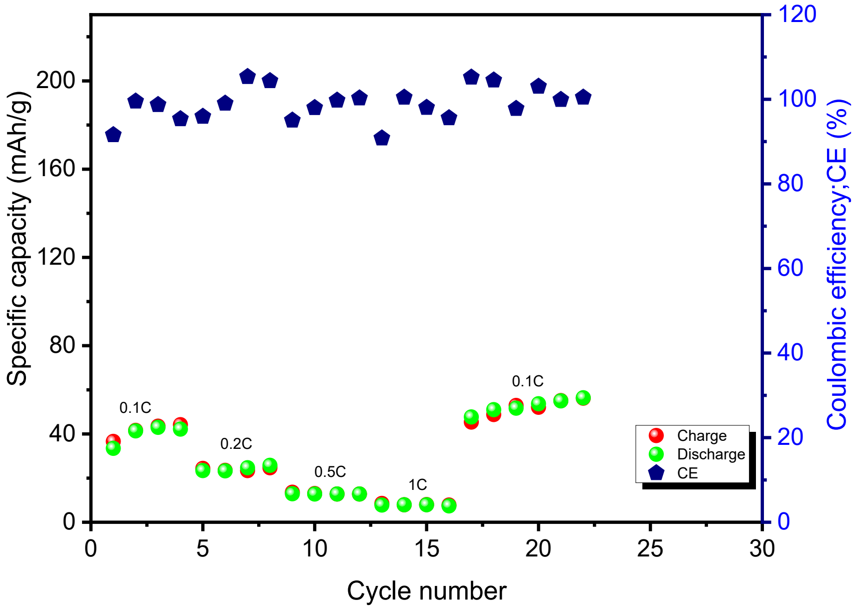

Figure S6. Specific capacity and Coulombic efficiency vs. cycle number for the Li°|T1-70|LFP cells at 80 °C.

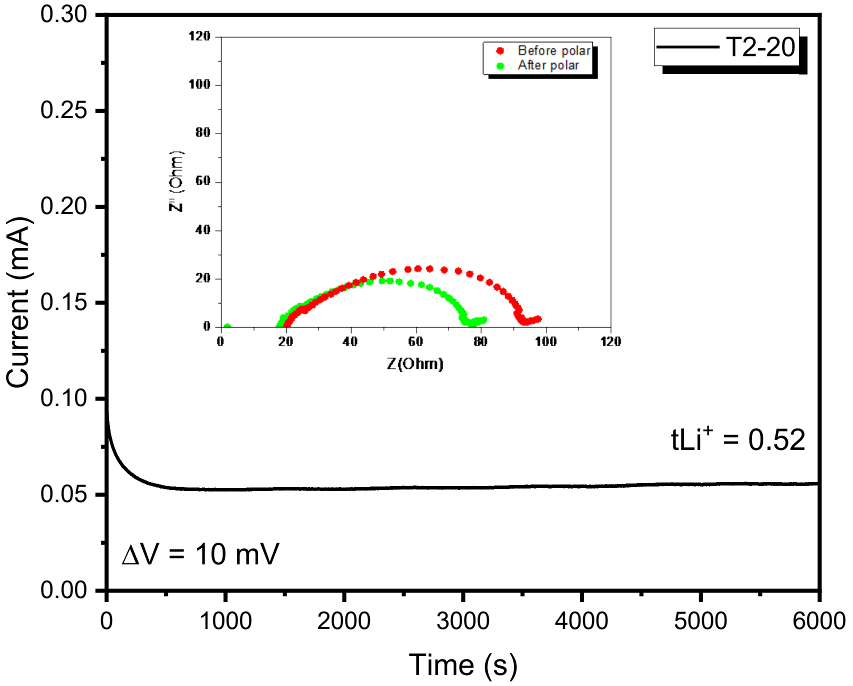

Figure S7. Chronoamperometry profile of the symmetric Li°|T2-20|Li° (inset shows the EIS curves before and after polarization).

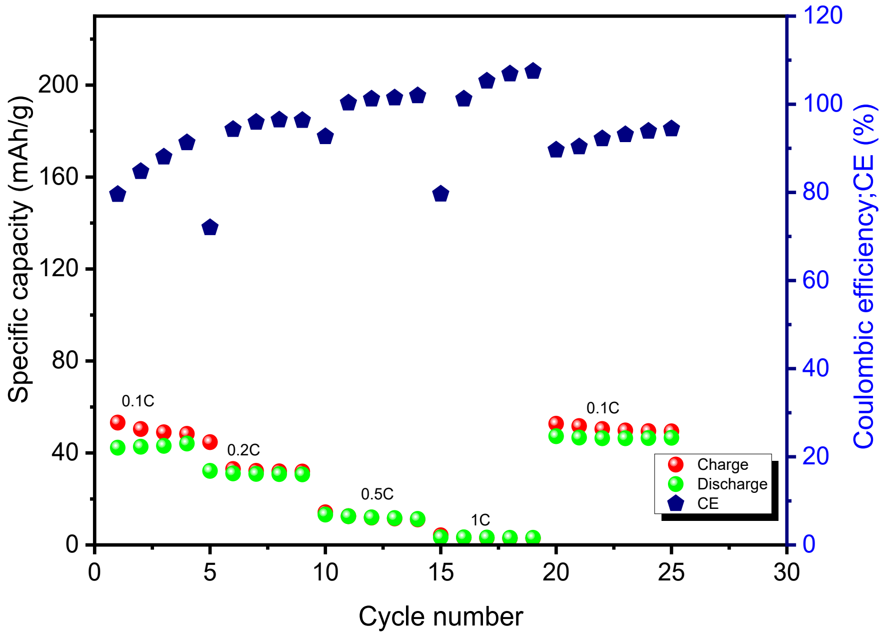

Figure S8. Specific capacity and Coulombic efficiency vs. cycle number for the Li°|T2-20|$LiFePO_4$ cells at 80 °C.

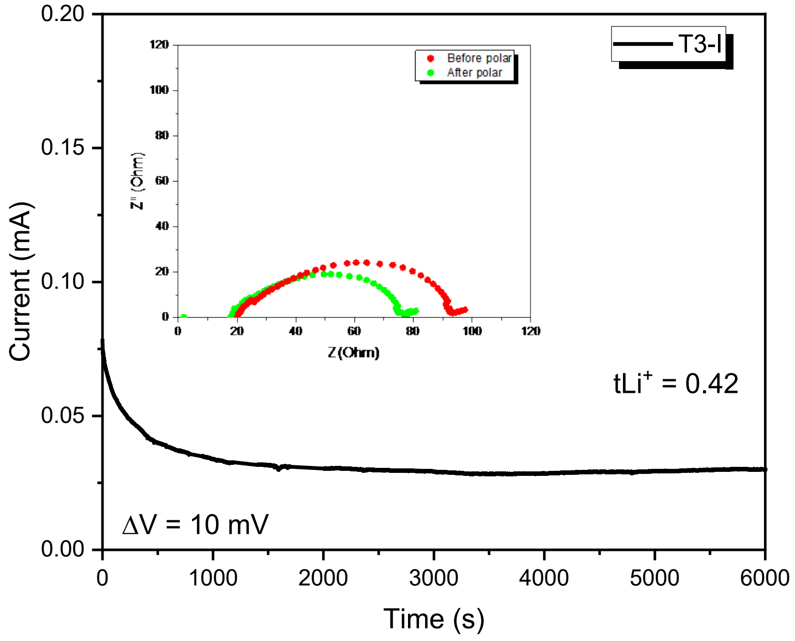

Figure S9. Chronoamperometry profile of the symmetric Li|T3-I|Li (inset shows the EIS curves before and after polarization).
